# Optical Coherence Tomography of Retinal Degeneration in Royal College of Surgeons Rats and Its Correlation with Morphology and Electroretinography

**DOI:** 10.1371/journal.pone.0162835

**Published:** 2016-09-19

**Authors:** Kobu Adachi, Shizuka Takahashi, Kodai Yamauchi, Natsuki Mounai, Reiko Tanabu, Mitsuru Nakazawa

**Affiliations:** Department of Ophthalmology, Hirosaki University Graduate School of Medicine, 5 Zaifu-cho, Hirosaki, 036–8562, Japan; University of Florida, UNITED STATES

## Abstract

**Purpose:**

To evaluate the correlation between optical coherence tomography (OCT) and the histological, ultrastructural and electroretinography (ERG) findings of retinal degeneration in Royal College of Surgeons (RCS^-/-^) rats.

**Materials and Methods:**

Using OCT, we qualitatively and quantitatively observed the continual retinal degeneration in RCS^-/-^ rats, from postnatal (PN) day 17 until PN day 111. These findings were compared with the corresponding histological, electron microscopic, and ERG findings. We also compared them to OCT findings in wild type RCS^+/+^ rats, which were used as controls.

**Results:**

After PN day 17, the hyperreflective band at the apical side of the photoreceptor layer became blurred. The inner segment (IS) ellipsoid zone then became obscured, and the photoreceptor IS and outer segment (OS) layers became diffusely hyperreflective after PN day 21. These changes correlated with histological and electron microscopic findings showing extracellular lamellar material that accumulated in the photoreceptor OS layer. After PN day 26, the outer nuclear layer became significantly thinner (*P* < 0.01) and hyperreflective compared with that in the controls; conversely, the photoreceptor IS and OS layers, as well as the inner retinal layers, became significantly thicker (*P* < 0.001 and *P* = 0.05, respectively). The apical hyperreflective band, as well as the IS ellipsoid zone, gradually disappeared between PN day 20 and PN day 30; concurrently, the ERG a- and b-wave amplitudes deteriorated. In contrast, the thicknesses of the combined retinal pigment epithelium and choroid did not differ significantly between RCS^-/-^ and RCS^+/+^ rats.

**Conclusion:**

Our results suggest that OCT demonstrates histologically validated photoreceptor degeneration in RCS rats, and that OCT findings partly correlate with ERG findings. We propose that OCT is a less invasive and useful method for evaluating photoreceptor degeneration in animal models of retinitis pigmentosa.

## Introduction

Retinitis pigmentosa (RP) is the most common hereditary retinal photoreceptor degenerative disease; more than 60 mutated genes have been identified that can cause RP (RetNet^™^, Retinal Information Network: https://sph.uth.edu/retnet/home.htm). In addition, RP is one of the main causes of legal blindness, and the overall incidence is 1 in 4,000–5,000 people worldwide. The *Mertk* gene, which encodes a receptor tyrosine kinase, causes autosomal recessive RP [1]. Among Japanese cases of RP whose causative gene mutations have been identified, it is estimated that 2.5% are caused by the *Mertk* gene mutations [2]. Interestingly, a mutation in this gene was originally found in Royal College Surgeons (RCS) rats that demonstrate hereditary retinal dystrophy [3]. Therefore, dystrophic RCS rats (RCS^-/-^) can be regarded as an animal model of the human autosomal recessive form of RP that is associated with the *Mertk* gene mutations. Specifically, a defective *Mertk* gene prevents the retinal pigment epithelium (RPE) from phagocytizing and shedding the photoreceptor outer segments (OSs). This eventually leads to photoreceptor degeneration [1, 3]. Relatedly, Dowling and Sidman reported that the extracellular lamellar material accumulates in the photoreceptor OS layer in RCS^-/-^ rats [4]. This is also likely caused by defective phagocytosis of the OS, which is induced by the *Mertk* gene mutation. In addition, since the *Mertk* gene is specifically expressed in the RPE, transfer of the wild type *Mertk* gene into the RPE can rescue the photoreceptor degeneration [5, 6]. In fact, trials of gene therapy have already started in patients with *Mertk*-associated RP [7]; for this reason, this particular type of RP has attracted increasing scientific attention.

Recent advances in optical coherence tomography (OCT) technology have provided previously unknown morphologic detail—particularly in the central area of the eyeball; this has allowed improved clinical evaluation of RP [8–21]. Because OCT is non-invasive and can be repeated several times, it confers a significant advantage to investigators analyzing the morphological changes that accompany disease progression in animal models of RP. In addition, OCT provides *in vivo* images without the potential artifacts that are caused by histological processing [22]. The technique may also become useful in morphologically evaluating how therapeutic drugs protect the photoreceptor. Previous investigators have evaluated retinal degeneration using OCT in P23H heterozygous rhodopsin transgenic rats, in retinal degeneration (rd) 10 and rd12 mice, and in arrestin-1 knock-out mice was reported in detail [22–26]. However, in RCS^-/-^ rats, OCT has rarely been used to evaluate retinal morphology.

RCS^-/-^ rats are an important animal model of RP; they exhibit unique phenotypic features and distinct molecular mechanisms. Therefore, a detailed understanding of the morphology of retinal degeneration in RCS^-/-^ rats—as imaged using OCT—will improve our understanding of the photoreceptor degeneration associated with the *Mertk* gene mutations. In this study, we attempted to characterize retinal degeneration in RCS^-/-^ rats through chronological examination using OCT, and by comparing the OCT findings with the corresponding histological, electron microscopic, and electroretinography (ERG) findings. In clinical practice, we usually carry out cross-sectional OCT and ERG in patients with RP; however, it is difficult to ascertain the relationship between the OCT and ERG findings, because the patients have heterogeneous genetic and clinical features. Models of RP, such as the RCS^-/-^ rats, provide chronological information on morphology from OCT findings, with corresponding ERG and histological changes that are associated with a single genotype. We believe that these findings will be useful in evaluating the relationship or correlation between the morphologic and functional changes that occur *Mertk* mutation-associated retinal degeneration.

## Materials and Methods

### Experimental Animals

All experimental procedures conformed with the Association for Research in Vision and Ophthalmology (ARVO) Statement for the Use of Animals in Ophthalmic and Vision Research, and were approved by the Committee of Ethics in animal experiments of Hirosaki University Graduate School of Medicine (Approval Number: M11026). RCS^-/-^ rats and control, wild-type RCS^+/+^ rats were obtained from Japan Clea (Tokyo, Japan). All the RCS rats had an albino background. The animals were maintained at the Hirosaki University Graduate School of Medicine animal care service facility under a cycle of 12 hours light (50 lx illumination in the cage) and 12 hours darkness (< 10 lx environmental illumination). Care was taken not to cause light-induced photoreceptor damage. Food and water were available *ad libitum*.

### OCT Examination

**Fig 1:**
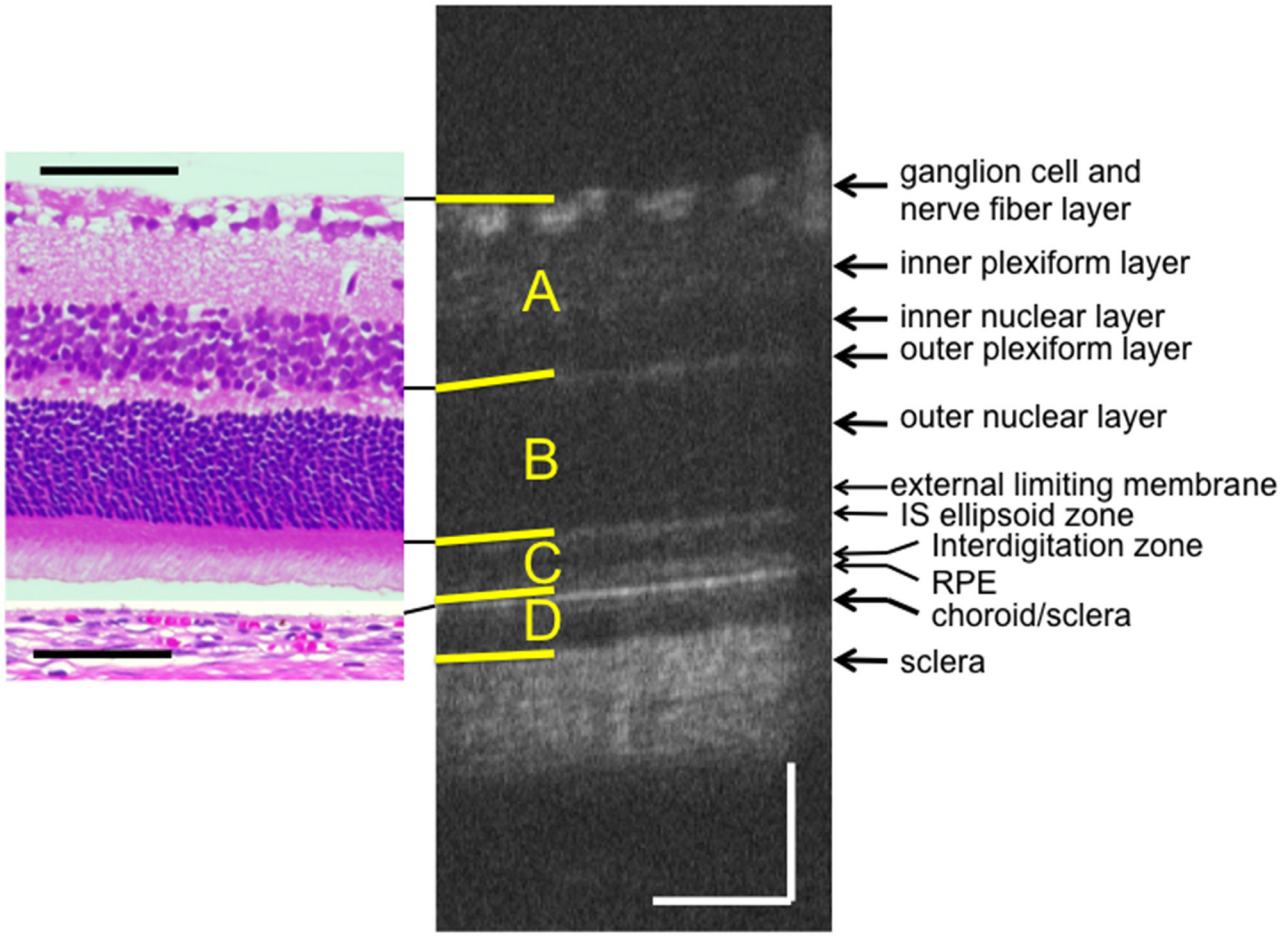
Typical OCT image of a RCS^+/+^ rat at PN day 33, along with a corresponding histological section of a RCS^+/+^ rat at PN day 29 (hematoxylin and eosin stained). The retinal pigment epithelium (RPE) was detached. The top yellow line indicates the retinal surface, the second line indicates the border between the inner nuclear layer and outer plexiform layer, the third line indicates the upper limit of the IS ellipsoid line, the fourth line indicates the surface of the RPE, and the bottom line indicates the bottom of the choroid. Layer A comprises the nerve fiber layer, the ganglion cell layer, the inner plexiform layer and the inner nuclear layer. Layer B comprises the outer plexiform layer and the outer nuclear layer. Layer C comprises the photoreceptor IS and OS layers. Layer D comprises the RPE and the choroid. The black and white bars represent 100μm length.

OCT was performed using a Micron^®^ IV (Phoenix Research Labs, Pleasanton, CA) with a contact lens specifically designed for rat OCT. In the RCS^-/-^ rats, OCT was conducted at 12 time points between postnatal (PN) day 17 and PN day 111, while in the RCS^+/+^ rats, OCT was carried out at eight time points between PN day 18 and PN day 67. At each time point, four to eight rats (eight to 16 eyes) were evaluated. The rats were anesthetized by intraperitoneal injection of a mixture of medetomidine hydrochloride (0.315mg/kg), midazolam (2.0mg/kg), and butorphanol tartrate (2.5mg/kg). To alleviate the pain associated with injection, the rats were pre-anesthetized by inhalation of 80% carbon dioxide and 20% oxygen prior to the intraperitoneal injection. The researchers monitored the physical conditions of the rats including heart beat and respiratory pattern, by inspection and gentle palpation every minute during the experiment. The pupils were dilated using eyedrops that contained a mixture of 0.5% tropicamide and 0.5% phenylephrine hydrochloride. The corneal surface was protected using a 1.5% hydroxyethylcellulose solution. The rat ocular fundus was monitored using the fundus camera of the Micron^®^ IV, and the position of the retinal OCT image was set horizontally at one disc diameter superior to the optic disc. Fifty images were averaged to eliminate projection artifacts. The acquired OCT images were quantitatively analyzed using the InSight^®^ software (Phoenix Research Labs). Five images from five rats in each genotype group were selected at each time point on the basis of image sharpness; importantly to avoid selection bias, the pictures were not selected by thickness or reflectivity. We measured the thicknesses of the inner (A, Fig 1), middle (B, Fig 1), and outer (C, Fig 1) layers of the neural retina, as well as that of the combined RPE and choroid (D, Fig 1). The middle layer consists of the combined outer plexiform and outer nuclear layers, and the outer layer consists of the photoreceptor inner segment (IS) and OS layers (Fig 1).

### Analysis of Retinal Layer Thickness

Using the InSight^®^ software, the borderlines between the retinal sublayers (A-D) were defined on the OCT pictures. These borderlines were initially indicated automatically by the InSight^®^, they were then manually corrected by the researchers if necessary. Next, the distance (in μm) between each borderline was calculated using InSight^®^ at 10 different points that were evenly distributed throughout the borderline, and the average of these data was defined as the thickness of the respective sublayer. The overall average retinal layer thickness was presented as mean ± standard deviation; it was calculated from the values obtained from five different OCT images of five different eyes in rats of the same age.

### ERG Examination

Scotopic full-field ERGs were recorded at 10 time points between PN day 17 and PN day 53 in the case of RCS^-/-^ and at 6 time points between PN day 15 and PN day 52 in the case of RCS^+/+^ rats. Rats were dark-adapted for at least 24 hours; they were then anesthetized following the same method described above. The physical conditions were also monitored using the methods described above. A reference electrode was placed in the center of the scalp, and a ground electrode was placed in the proximal portion of the tail skin. During the measurement, the body temperature was maintained at 37°C using a body warmer. Pupils were dilated using eyedrops composed of a mixture of 0.5% tropicamide and 0.5% phenylephrine hydrochloride. After the corneal surface had been anesthetized using 0.4% oxybuprocaine hydrochloride eyedrops, a contact-lens electrode (Micron^®^ Ganzfeld ERG, Phoenix Research Labs) was applied directly to the corneal surface. In accordance with the standards of the International Society for Clinical Electrophysiology of Vision [27], light stimulus intensities were set at 3.0, 10.0 and 30.0 cd.s/m^2^. We acquired 20 responses at 3.0 cd.s/m^2^ (stimulus interval = 10 s) and 10 responses each at 10.0 and 30.0 cd.s/m^2^ (stimulus interval: 20 s); these responses were then averaged to create a standard waveform. The amplitudes of both a- and b-waves (n = 3–6 at each time point) were statistically analyzed.

### Histological Examination

Histological examinations were performed using eyes excised from RCS^-/-^ and RCS^+/+^ rats between PN day 17 and PN day 53. Immediately after euthanasia by carbon dioxide inhalation, the eyeballs were enucleated under a microscope and fixed in 4% paraformaldehyde solution (pH 7.0) for at least 24 hours at 4°C. The eyes were embedded in paraffin and cut into 6-μm thick sections; this sectioning was carried out horizontally at the level of one disc diameter superior to the optic disc to ensure the position was identical to that of the OCT measurement. The excised tissue was stained using hematoxylin and eosin (HE) and was photographed under a light microscope (DP-71; Olympus, Japan). The histological findings were compared to the corresponding findings from the OCT images.

### Electron Microscopic Examination

Electron microscopic examinations were performed using eyes excised from RCS^-/-^ rats between PN day 18 and PN day 28. Immediately after enucleation by the methods described above, the eyes were fixed in a solution of 2.5% glutaraldehyde and 2% paraformaldehyde solution (pH 7.4) for 24 hours at 4°C. The retina and choroid were excised, post-fixed in phosphate buffered 1% osmium tetroxide (pH 7.4), dehydrated in an ascending series of ethanol solutions, and passed through propylene oxide. The blocks were embedded in epoxy resin. Thin sections were stained using uranyl and lead salt solutions. The sections were photographed using transmission electron microscopy (Filgen^®^, Nagoya, Japan).

### Statistic Examination

All the statistical calculations were performed using the SPSS software version 22 (Statistical Package for the Social Sciences, Chicago, IL). Data from the two groups were compared using ANOVA, after normality of distribution had been confirmed using the Shapiro-Wilk test. *Post hoc* analyses were performed using Bonferroni’s test. *P*-values < 0.05 were considered statistically significant.

## Results

### OCT Findings in RCS^+/+^ Rats

**Fig 2:**
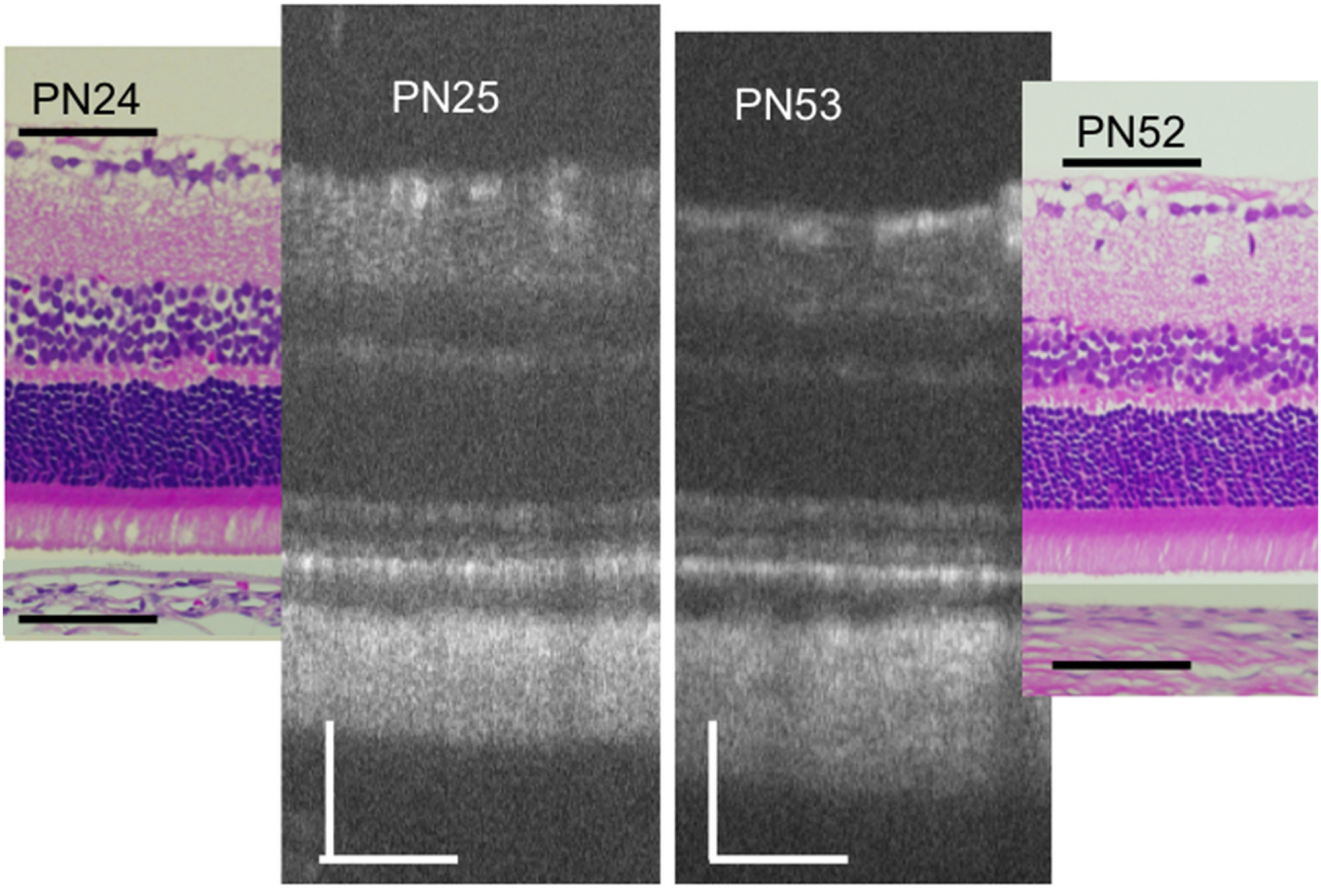
Characterization of typical OCT findings from RCS^+/+^ rats at PN days 25 and 53, as well as the corresponding histological sections from RCS^+/+^ rats at PN days 24 and 52 (hematoxylin and eosin stained, the retinal pigment epithelium was detached.). The black and white bars represent 100μm length.

Fig 1 shows a typical OCT image of a PN day 32 RCS^+/+^ rat, as well as the corresponding image of an HE-stained histology section of an RCS^+/+^ rat at PN day 29. The OCT images of the RCS^+/+^ rats indicated that the structure of the retina was consistent throughout the observation period (PN days 18–67). On the same note, Fig 2 demonstrates that the OCT and histological findings on PN days 24–25 were similar to those on PN days 52–53 (original images are presented in S1 Fig). In RCS^+/+^ rats, the following structures were clearly identified in the OCT images: the ganglion and nerve fiber layers, inner plexiform layer, inner nuclear layer, outer plexiform layer, outer nuclear layer, photoreceptor IS and OS layers, RPE layer, choroid, and sclera. In addition, regarding the detailed structure of the photoreceptor IS and OS layers, two distinct hyperreflective bands—corresponding to the IS ellipsoid zone and the interdigitation zone—were constantly visualized in the RCS^+/+^ rat OCT images (Fig 1). These zones have been defined in the human foveal OCT images [28], as well as in previous reports involving mouse OCT [22, 25]. However, the external limiting membrane was sometimes difficult to identify. The layers observed in the OCT images were confirmed in the corresponding histological sections (Figs 1 and 2). The observed discrepancy in retinal thickness between the OCT and histological images may be partly due to artifacts introduced during tissue fixation and/or sectioning.

### Qualitative Analyses of OCT Findings from RCS^-/-^ Rats

**Fig 3:**
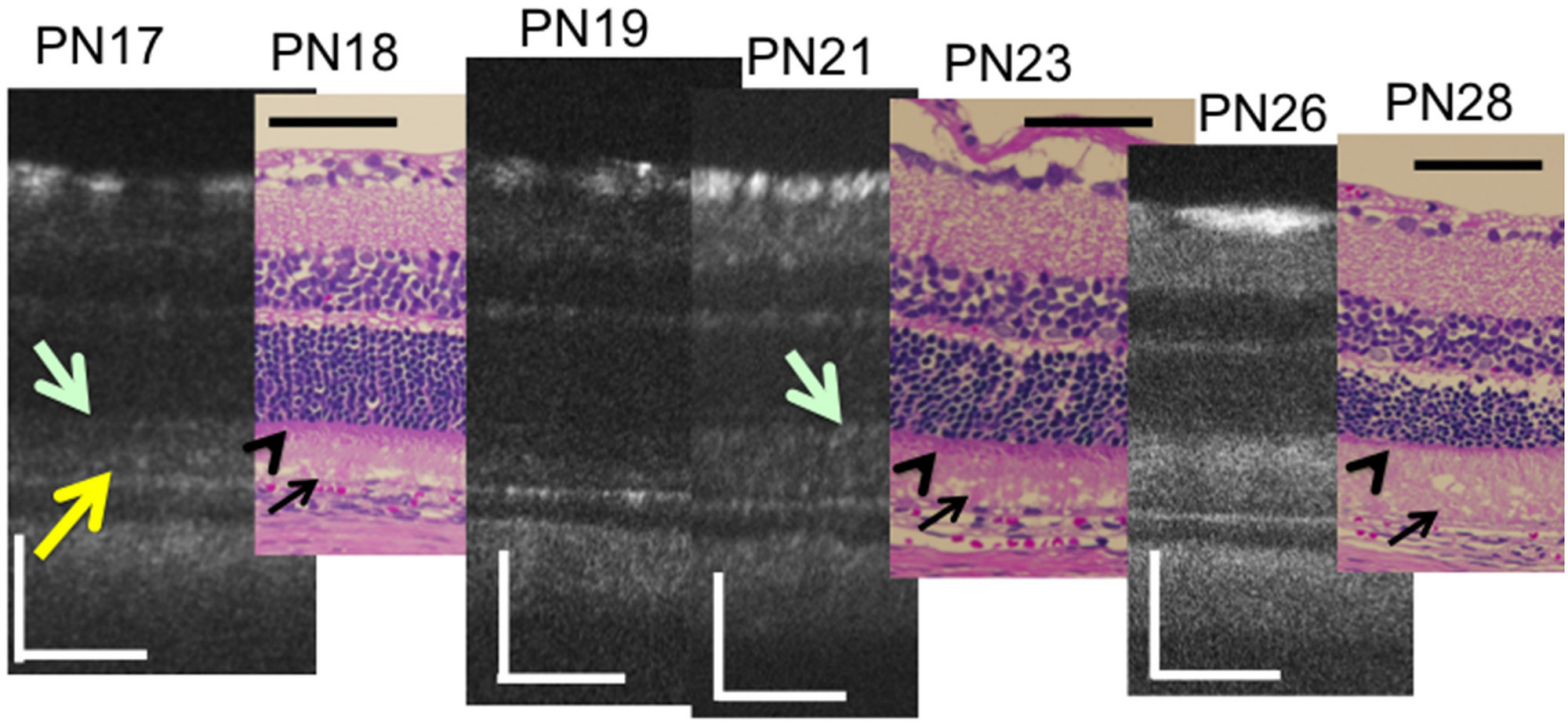
Chronological changes in the photoreceptor IS and OS layers during the early stages (PN days 17 to 26) of retinal degeneration in RCS^-/-^ rat—observed using optical coherent tomography and the corresponding histological sections (PN days 18, 23, and 28). A yellow arrow indicates the apical hyperreflective band and a light green arrow indicates the IS ellipsoid zone. Black arrows indicate the extracellular lamellar material. Arrowheads indicate the photoreceptor inner segment. The black and white bars represent 100μm length.

**Fig 4:**
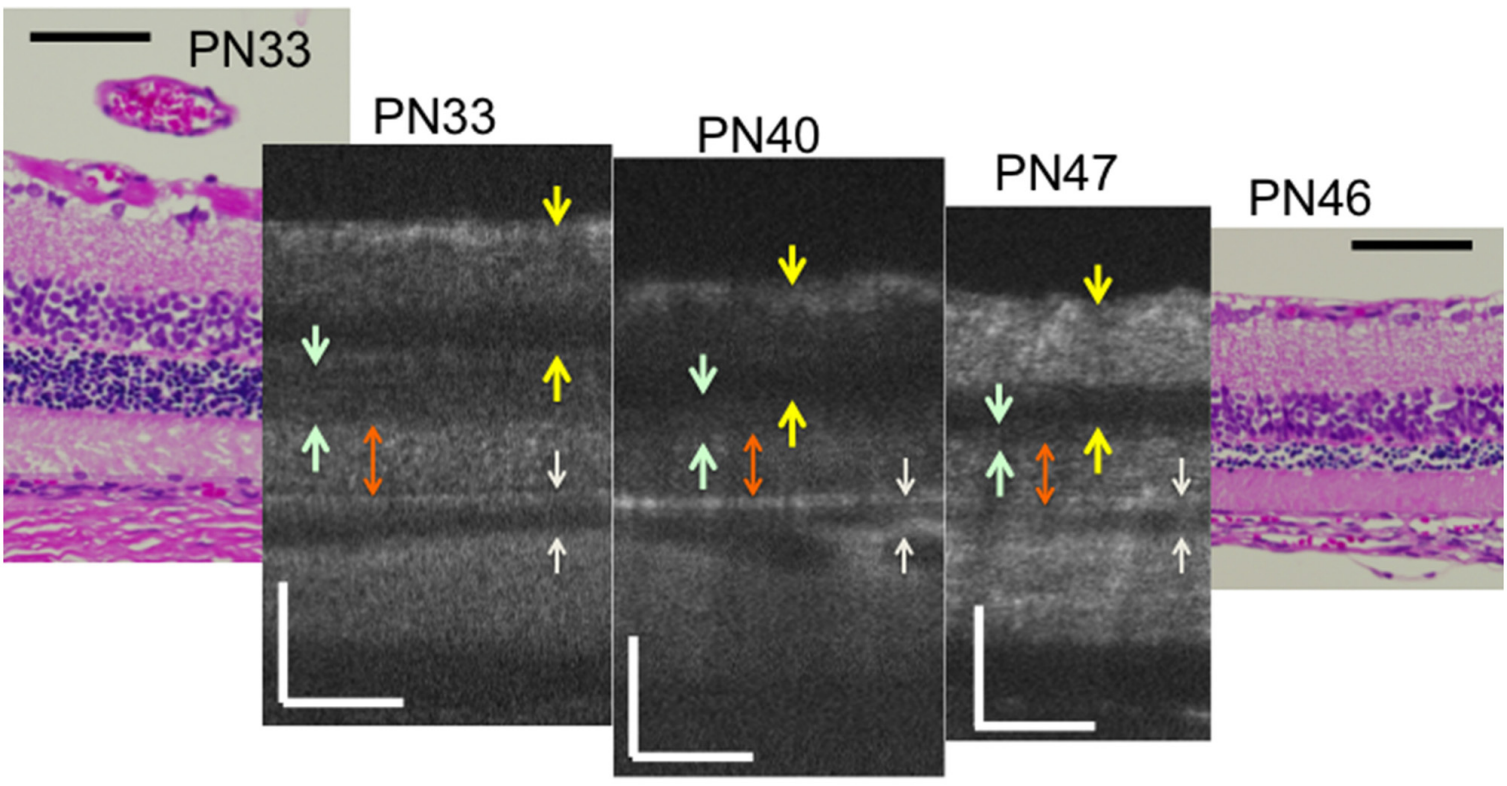
Chronological changes in the retinal layers during the progressive stage (PN days 33 to 47) of RCS^-/-^ rat retinal degeneration—observed using optical coherent tomography and the corresponding histological sections (PN days 33 and 46). Dark green arrows indicate the outer nuclear layer. An orange arrow indicates the photoreceptor IS and OS layers. Yellow arrows indicate the inner retinal layer. White arrows indicate the retinal pigment epithelium and choroid. The black and white bars represent 100μm length.

**Fig 5:**
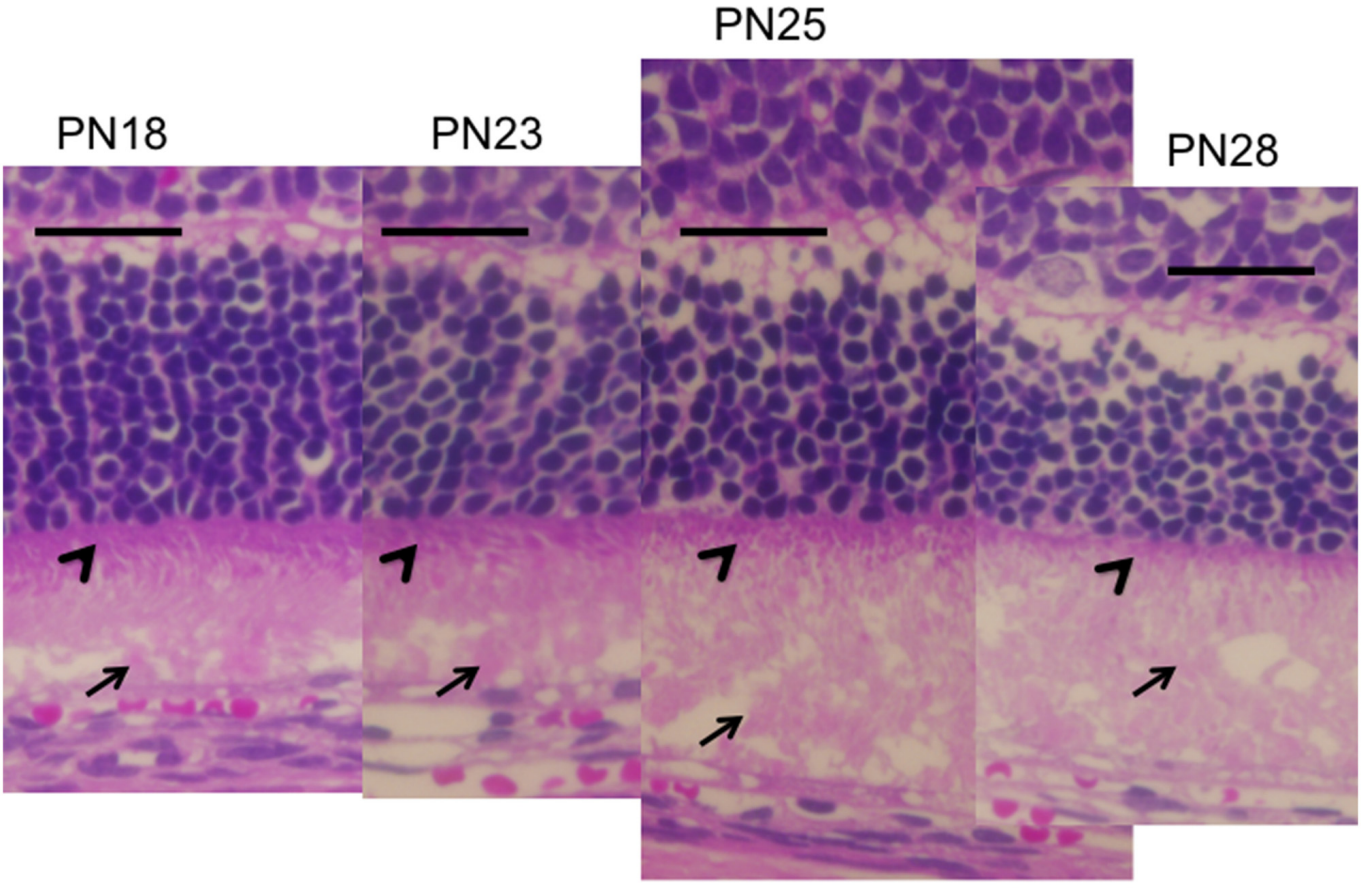
Chronological histological changes in the photoreceptor layer during the early stages (PN days 18 to 28; hematoxylin and eosin stained) of retinal degeneration in RCS^-/-^ rats. Arrows indicate the extracellular lamellar material deposited on the apical surface of the RPE (PN days 18 and 23) and in the photoreceptor OS layer (PN days 25 and 28). Arrowheads indicate the photoreceptor IS layer (PN days 18 to 28). The black bar represents 50μm length.

**Fig 6:**
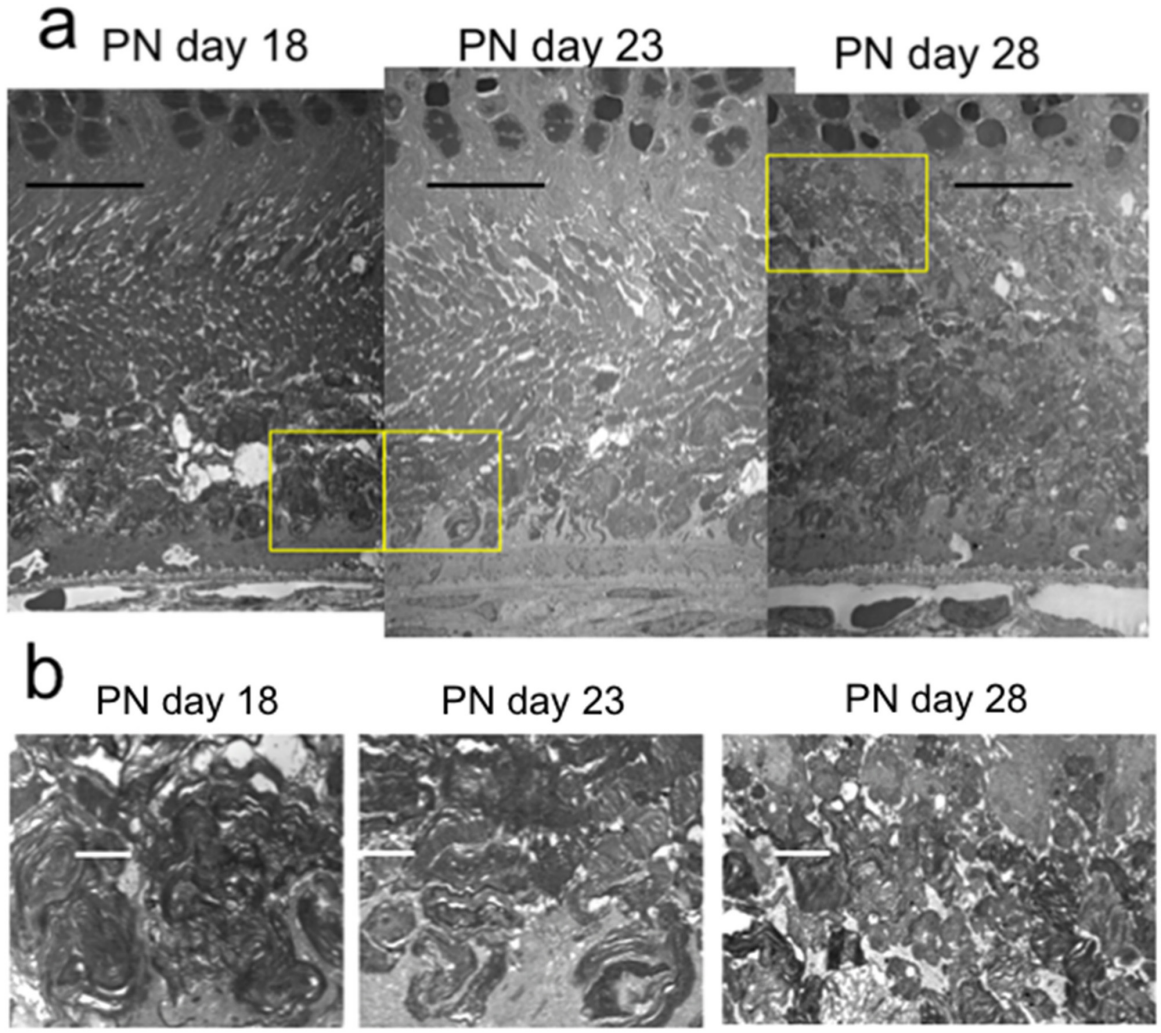
Electron microscopic findings of the photoreceptor IS and OS layers of RCS^-/-^ rats at PN days 18, 23, & 28. The semiround-shaped deposits of extracellular lamellar material had accumulated at the apical portion of the OS at PN days 18 and 23 (a). These deposits gradually increased toward the basal side of the OS until PN day 28 (a). The magnified picture of each yellow box is presented in the lower level, exhibiting the lamellar structure of the deposited material (b). The black bar represents 10μm, and the white bar represents 2μm length, respectively.

**Fig 7:**
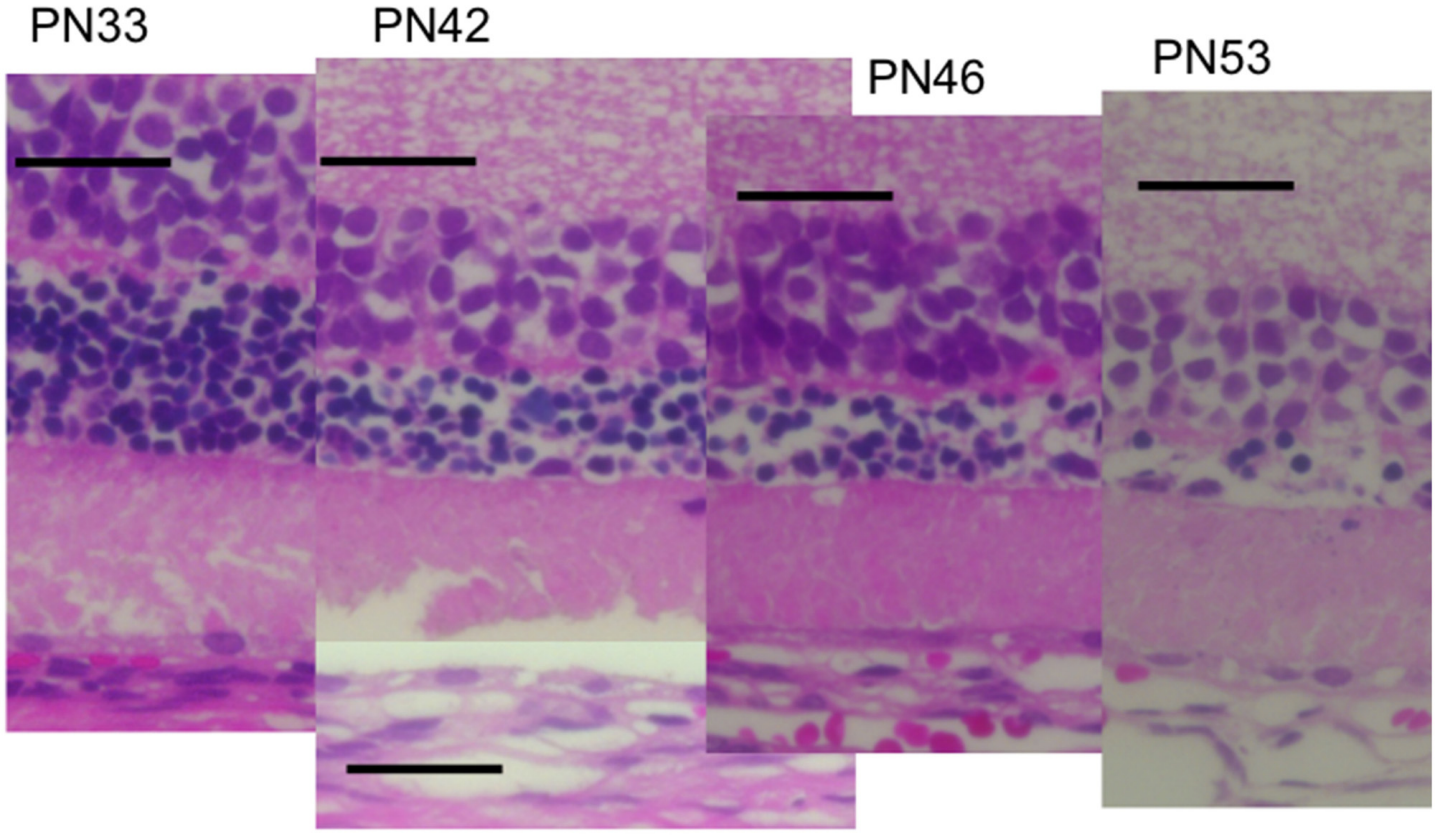
Chronological histological changes in the photoreceptor layer during the progressive stage (PN days 33 to 53; hematoxylin and eosin stained) of retinal degeneration. The RPE had detached at PN day 42. The extracellular lamellar material occupied the photoreceptor IS and OS layers after PN day 33. The nuclei of the outer nuclear layer had become pycnotic. The black bar represents 50μm length.

Similarly, we analyzed the OCT findings of RCS^-/-^ rats to identify characteristic changes that occur during retinal degeneration. Figs 3 and 4 show characteristic OCT images of RCS^-/-^ rats. During the initial stage of degeneration, at PN days 17 and 19 the inner and outer retinal layers appear to be similar to those in the wild type retina. For instance, there were two distinct hyperreflective bands in the photoreceptor IS and OS layers (Fig 3; original images are presented in S2 Fig). The band at the basal side of the IS and OS layers corresponded to the IS ellipsoid zone, as determined by comparison with the histological findings. However, although the hyperreflective band at the apical side of the OS layer appeared similar to the interdigitation zone in terms of position, it seemed to be slightly broader in RCS^-/-^ rats than in wild type rats (Figs 1 and 2). Furthermore, when it was compared with the histological and ultrastructural findings, this band appeared to correspond to the aforementioned extracellular lamellar material that accumulates in the OS layer of the RCS^-/-^ rats [4] (Figs 3, 5 and 6). For ease of reference, we dubbed this hyperreflective band observed at the apical side of the photoreceptor OS the “apical hyperreflective band”; we presumed that it corresponded to the extracellular lamellar material [4]. Subsequently, at PN day 21, the IS ellipsoid zone became obscure, and the photoreceptor IS and OS layers themselves began to become hyperreflective. At PN day 26, the apical hyperreflective band was completely unidentifiable, as was the IS ellipsoid zone; instead, the IS and OS layers were diffusely hyperreflective (Fig 4; original images are presented in S3 Fig). These qualitative changes in the photoreceptor IS and OS layers—observed using OCT—corresponded to histological findings indicating that the deposition of extracellular lamellar material had progressed from the apical to the basal side of the photoreceptor OS layer, and that the IS layer had narrowed (Fig 5) [4]. In electron micrographic findings, the lamellar structure of semiround-shaped extracellular lamellar material that was deposited at the apical portion of the OS layer at PN days 18 and 23 was demonstrated, as well as the absence of the interdigitation structure that is normally found in the OS layer (Fig 6). In addition, at PN day 28 the degenerated lamellar material was observed even at the basal portion of the OS layer (Fig 6b). During the following progressive stage, at PN day 33 the outer nuclear layer started to thin; by PN day 47 it had become extremely thin (Figs 4 and 7). In addition, the photoreceptor nuclei in the outer nuclear layer had become pycnotic at PN day 33 (Fig 7), and the corresponding OCT images revealed that the outer nuclear layer had become hyperreflective and thinner than at PN days 21 to 26 (Figs 3 and 4). Conversely, the inner nuclear layer showed constant hyperreflectivity between PN days 17 and 47; indeed, this finding was used to normalize our data. These changes in the outer nuclear layer progressed rapidly; in contrast, the thicknesses of the inner retinal layer, the photoreceptor IS and OS layers, and the RPE and choroid were apparently consistent (Figs 3 and 4). Finally, after PN day 54, the outer nuclear layer had become unidentifiable under OCT (data not shown).

### Quantitative (Retinal Thickness) Analyses of OCT Findings

**Fig 8:**
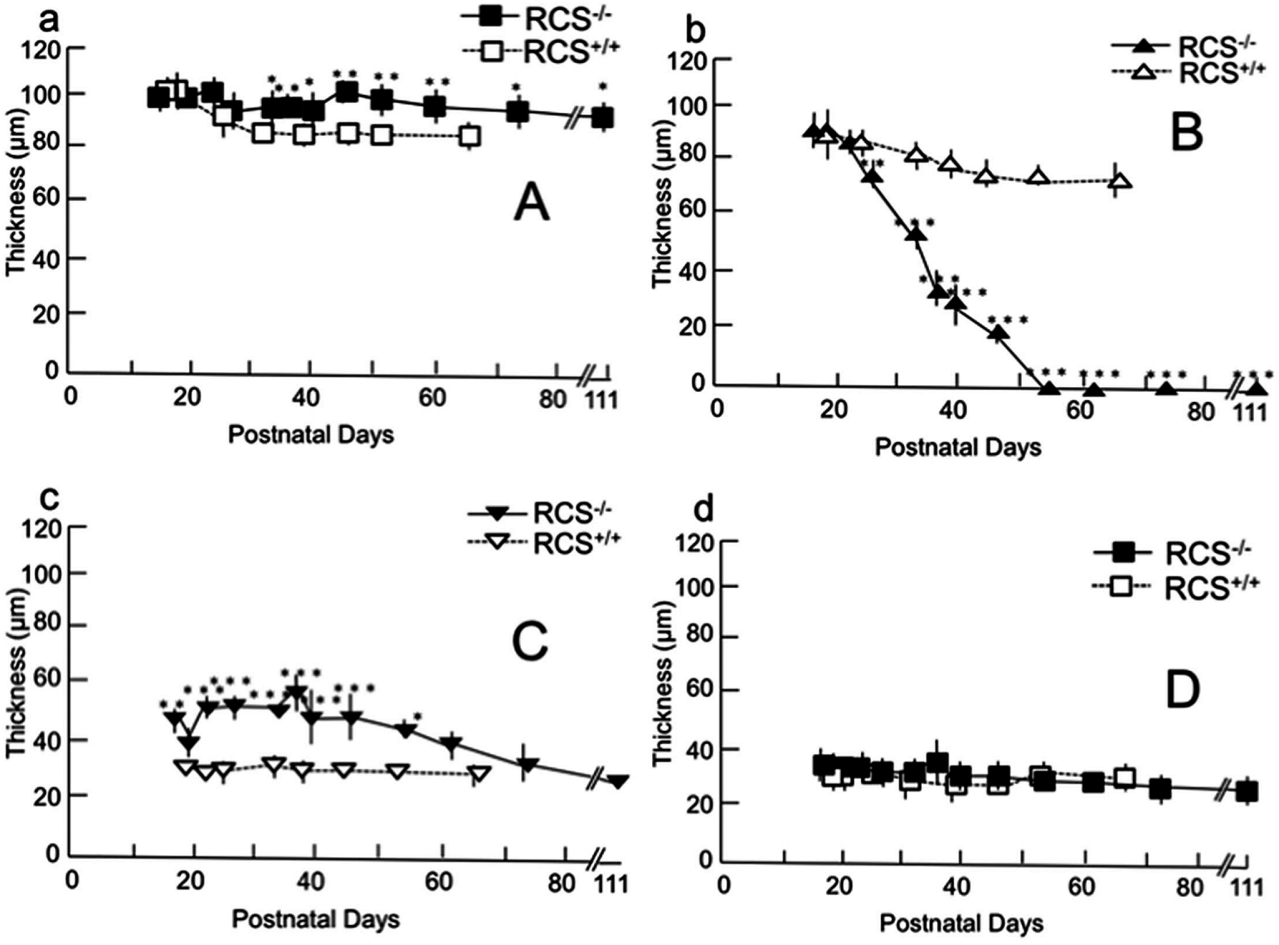
Chronological changes in the thickness of each retinal sublayer (μm), A (a), B (b), C (c), and D (d). Layer A comprises the nerve fiber layer, the ganglion cell layer, the inner plexiform layer and the inner nuclear layer. Layer B comprises the outer plexiform layer and the outer nuclear layer. Layer C comprises the photoreceptor inner and outer segments layer. Layer D comprises RPE and the choroid. Closed squares (a and d) and triangles (b and c) indicate RCS^-/-^ rats and open squares (a and d) and triangles (b and c) indicate RCS^+/+^ rats. Bars indicate standard deviation. Statistical significance (with Bonferroni’s *post hoc* test): * *P* < 0.05; ** *P* < 0.01; *** *P* < 0.001.

Fig 8 shows the time course of the change in thickness of each retinal layer in both RCS^-/-^ and RCS^+/+^ rats. The thickness of the inner retinal layer was significantly greater in the RCS^-/-^ than in the RCS^+/+^ rats after PN day 33 (Fig 8a; raw data are presented in S1 and S2 Tables). In contrast, the combined outer plexiform layer and outer nuclear layer in RCS^-/-^ rats underwent rapid thinning from PN day 26 to PN day 54 (*P* < 0.001); after PN day 54, this layer was no longer identifiable under OCT (Fig 8b). Meanwhile, the photoreceptor IS and OS layers in RCS^-/-^ rats were consistently significantly thicker than those in RCS^+/+^ rats between PN days 17 and 54 days (Fig 8c). The average thickness of the combined RPE and choroid did not differ significantly between the two groups, although there was a slight variation among animals and depending on the location of the retina imaged (Fig 8d).

### Correspondence of OCT Images and ERG Findings

**Fig 9:**
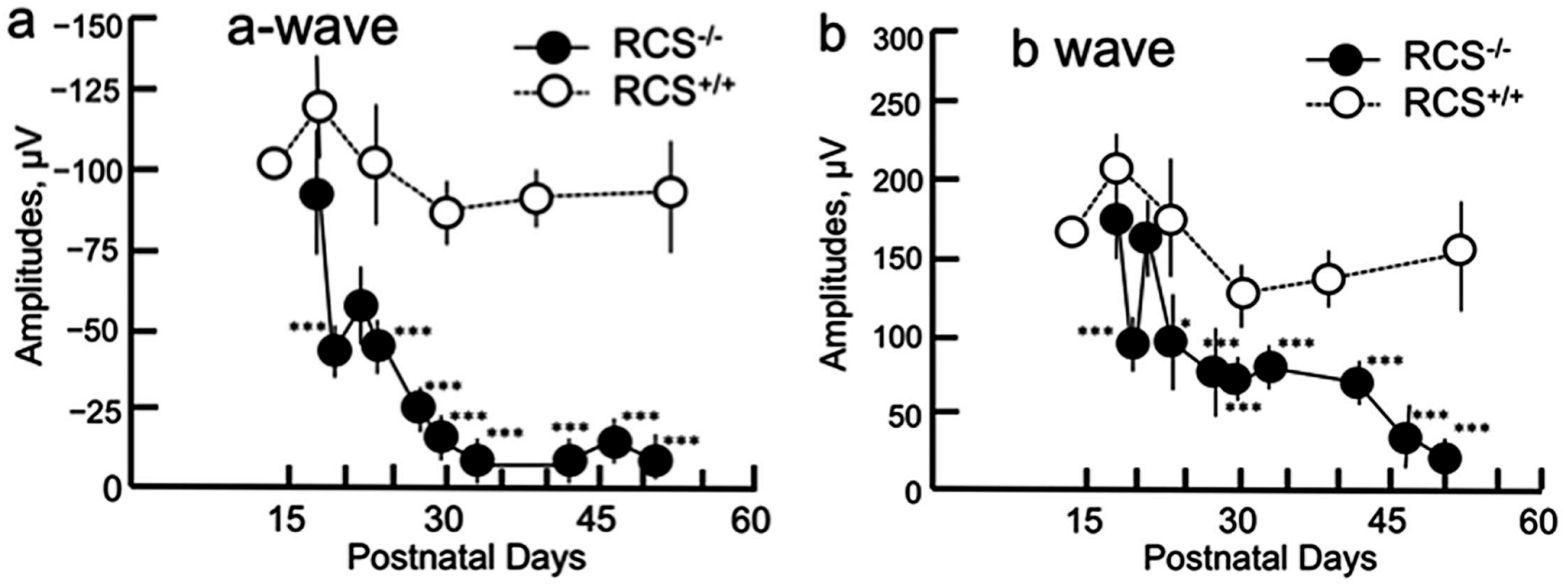
Chronological changes in the amplitudes of the ERG a- (a) and b- (b) waves in RCS rats. Closed circles indicate RCS^-/-^ rats and open circles indicate RCS^+/+^ rats. Statistical significance (with Bonferroni’s *post hoc* test): * *P* < 0.05; *** *P* < 0.001.

We confirmed that the amplitudes of both the a- and b-waves increased in a stimulus-dependent manner. For this reason, we employed the ERG data obtained using a light stimulus of 3.0 cd.s/m^2^ in the following analyses. Fig 9 shows the time courses of both the a- and b-waves in scotopic full-field ERG (3.0 cd.s/m^2^). At PN day 17 the amplitudes of both the a- and b-waves in RCS^-/-^ rats were slightly smaller than those of the wild type rats, although there was no statistical significance. Next, after PN day 19 both the a- and b-waves started to deteriorate, and there was a statistically significant decrease in their amplitudes. Particularly, the deterioration of the ERG amplitudes progressed rapidly after PN day 23 (Fig 9; raw data are presented in S3–S5 Tables). These changes appeared to correspond with the blurring and disappearance of the apical hyperreflective band and IS ellipsoid zone, as well as the hyperreflectivity of the photoreceptor IS and OS layers, that were observed during the same period under OCT. These findings also corresponded to the accumulation of extracellular lamellar material in the photoreceptor OS layer, which was verified in the histological and ultrastructural findings. Conversely, throughout the observation period, the amplitudes of the a- and b-waves remained consistent in RCS^+/+^ rats (*P* = 0.559 and 0.202, respectively; ANOVA).

## Discussion

The present study provides the first OCT evidence of degenerative processes in the photoreceptor IS and OS layers of RCS^-/-^ rats. We analyzed the OCT findings in RCS^-/-^ rats to ascertain the morphological changes that characterize retinal degeneration in these rats. In addition, we evaluated the correlation between these OCT findings and the changes observed using ERG. Using OCT, we found two distinct horizontal hyperreflective bands in the photoreceptor IS and OS layers at PN day 17 (Fig 3). The apically located band appeared to be similar to the interdigitation zone observed in the wild type rats (Figs 1 and 2). However, because of its similarity to previously reported histological and ultrastructural findings [4], as well as the absence of the interdigitation structure in the RCS^-/-^ rats (Fig 6) [4], we concluded that this apical hyperreflective band was different from the interdigitation zone, and that it corresponded to the accumulated extracellular lamellar material. In addition, we reasoned that the basally located band corresponded to the IS ellipsoid zone, because it was similar to the bands observed in the wild type rats and because it corresponded with histological findings that showed an intact ellipsoid zone (Fig 3). The earliest change observed in OCT occurred at PN days 17 to 19, when the apical hyperreflective band in the photoreceptor OS layer became blurred and subsequently disappeared. This was followed by the blurring and disappearance of the IS ellipsoid zone; the photoreceptor IS and OS layers became diffusely hyperreflective after PN day 21. The period during which these morphological changes occurred corresponded to the deterioration of both the a- and b- waves in ERG. In addition, these morphological changes demonstrated in OCT corresponded to the deposition of extracellular lamellar material, which was detected in the HE-stained sections and electron microscopic sections (Figs 2, 4 and 6). Because these changes in the apical hyperreflective band began simultaneously with the deterioration of the ERG amplitudes at around PN days 19–23, we postulate that the blurring and disappearance of the apical hyperreflective band constitute the first signs of a severe photoreceptor dysfunction. Indeed, it has been hypothesized that the phagosomal ability of the RPE is defective in RCS^-/-^ rats [3], and that the abnormally degraded OS may interfere with both the structure and function of the photoreceptor OS and IS. Moreover, as the extracellular lamellar material accumulates, the intact OS becomes shorter [4]. These experimental results may explain the association between the changes in the photoreceptor IS and OS layers and the deterioration of the ERG a-wave amplitude. In addition, the thinning of the outer nuclear layer started later than the deterioration of the ERG amplitudes, as did nuclear pyknosis in that layer. So, OCT findings may partly predict the functional aspects of retinal photoreceptor degeneration in RCS rats.

In the present study, we also measured the thickness of the inner and outer retinal layers, as well as that of the combined RPE and choroid. As retinal degeneration progressed, the total retinal thickness rapidly decreased in the RCS^-/-^ rats after PN day 40, because the outer retinal layer, particularly the outer nuclear layer, began to thin. Notably, the rapid thinning of the outer nuclear layer occurred much later than the deterioration of the ERG amplitudes (Figs 7b and 8), implying that retinal degeneration starts in the photoreceptor OSs in RCS rats, and that it takes several days for the subsequent photoreceptor cell death to be accelerated. In addition, it may be that the photoreceptor IS and OS layers in RCS^-/-^ rats are significantly thicker than those in the RCS^+/+^ rats because extracellular lamellar material accumulates in the OS layer of RCS^-/-^ rats (Fig 7c). This accumulation may in turn be caused by molecular defects that stem from mutations in the *Mertk* gene itself; in any case, they result in disturbed phagocytosis of the OS by the RPE. Abnormally accumulated material can make the photoreceptor IS and OS layers relatively thick by PN day 57; after PN day 60, the layers may gradually degrade in parallel with the degeneration of the outer nuclear layer (Fig 7c).

Conversely, the inner retinal layer was preserved in the RCS^-/-^ rats; indeed, it was significantly thicker in the RCS^-/-^ rats than in the RCS^+/+^ rats after PN day 30 (Fig 7a). In a previous report on preserving the inner retinal layer of human patients with RP [29], the surviving rod cells sprouted numerous neurites into the inner retinal layer in RP [30]. These abnormal neurites reportedly exhibited a positive immunohistochemical reaction to rhodopsin antibody [30]. Because mutations in the *Mertk* gene are common cause of RP in both RCS^-/-^ rats and humans, the same phenomenon may occur during the retinal degeneration of RCS^-/-^ rats; consequently, the inner retinal layer may become slightly thicker in RCS^-/-^ rats than in RCS^+/+^ rats. To confirm this possibility, it will be necessary to carry out an immunohistochemical study to detect rhodopsin-positive neurites in the inner retinal layer of RCS^-/-^ rats. Another possible explanation for the increased thickness is that retinal degeneration causes edema that originates from the deep retinal vessels of the inner retina. Furthermore, there was no significant difference in the thickness of the combined RPE and choroid between RCS^-/-^ and RCS^+/+^ rats until PN day 61. This implies that the defective *Mertk* gene has a negligible effect on the choroid layer.

In the previous OCT studies involving animal models of retinal degeneration, OCT has provided information regarding ultrastructural changes in the IS and OS layers. Specifically, changes in light scattering, which can be detected using OCT, start earlier than the thinning of the outer nuclear layer [25]. In addition, OCT measures the *in vivo* retinal layer thickness more precisely than histological sections [23, 24, 31–33]. In the rodent model, it has been reported that there are two hyperreflective bands in the IS and OS layers [22, 15]. In healthy humans, these bands represent the IS ellipsoid zone and the interdigitation zone, respectively [28]. We observed two similar bands in the photoreceptor IS and OS layers at PN days 17 and 19 in RCS^-/-^ rats. One band was located at the basal portion of the photoreceptor layer; it was clear that this band corresponded to the IS ellipsoid zone, because the OCT images of the RCS^-/-^ and RCS^+/+^ rats also had similar histological findings (Fig 3). The apical band appeared to be similar to the human interdigitation zone; however, we believe that it corresponds rather to the extracellular lamellar material that is deposited at the apical side of the photoreceptor OSs (Figs 3 and 5), as we discussed above. In addition, the changes observed in the OCT images of the photoreceptor IS and OS layers correlated with the simultaneous deterioration of the ERG amplitudes (Figs 3, 5 and 8). These results imply that the close correspondence between the structure and function can partly be analyzed by OCT. The present study also provides a standard for OCT features that accompany RCS retinal degeneration. These standard features may be useful in future experimental trials that use OCT to evaluate the effects of candidate photoreceptor protective drugs during retinal degeneration in RCS animal models. In addition, these OCT features could be applied clinically to evaluate the pathological changes that occur in the retina of patients with *Mertk* mutation-associated RP.

## Supporting Information

S1 Fig Original OCT images of RCS^+/+^ rats at PN days 25, 32, and 53, respectively. (TIF)

S2 Fig Original OCT images of RCS^-/-^ rats at PN days 17, 19, 21, and 26, respectively. (TIF)

S3 Fig Original OCT images of RCS^-/-^ rats at PN 33, 40, and 47, respectively. (TIF)

S1 Table Raw data of the retinal layer analyses 1 of both RCS^+/+^ and RCS^-/-^ (dystrophic) rats. Length: μm.
(PDF)

S2 Table Raw data of the retinal layer analyses 2 of both RCS^+/+^ and RCS^-/-^ (dystrophic) rats. Length: μm.
(PDF)

S3 Table Summary of raw data of ERG measurements of RCS^+/+^ rats. A-wave and B-wave: μV.
(PDF)

S4 Table Summary of raw data of ERG measurements of RCS^-/-^ rats. A-wave and B-wave: μV.
(PDF)

S5 Table Summary of ERG measurements of RCS^+/+^ and RCS^-/-^ rats and statistical analyses. A-wave and B-wave: μV.
(PDF)
