# Supplementary figures and images for: Optical Coherence Tomography of Retinal Degeneration in Royal College of Surgeons Rats and Its Correlation with Morphology and Electroretinography
Source: PLoS One. 2016 Sep 19;11(9):e0162835. doi: 10.1371/journal.pone.0162835 (PMC5028068; doi:10.1371/journal.pone.0162835)

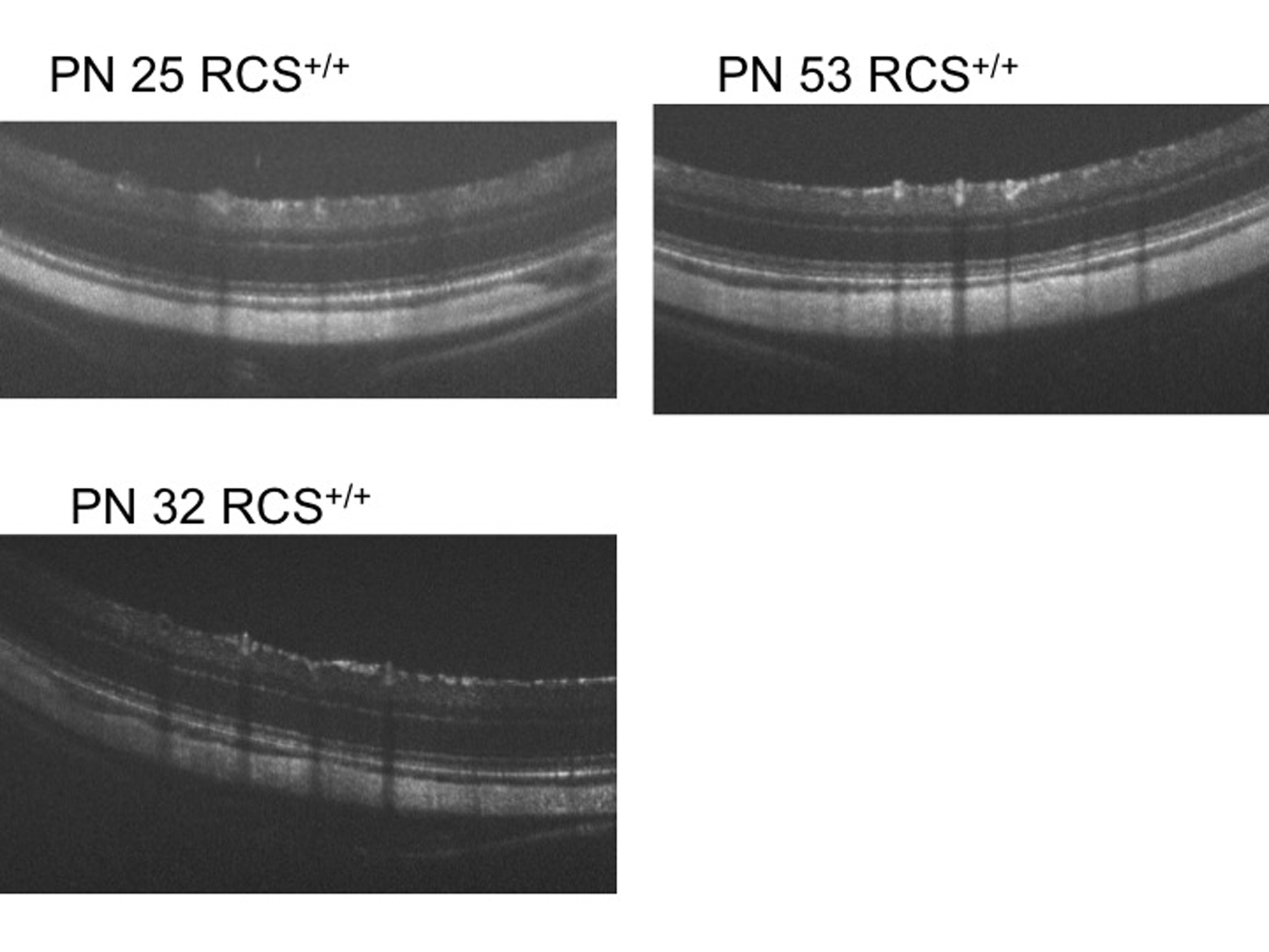

Supplement: S1 Fig — (TIF) [file pone.0162835.s001.tif]

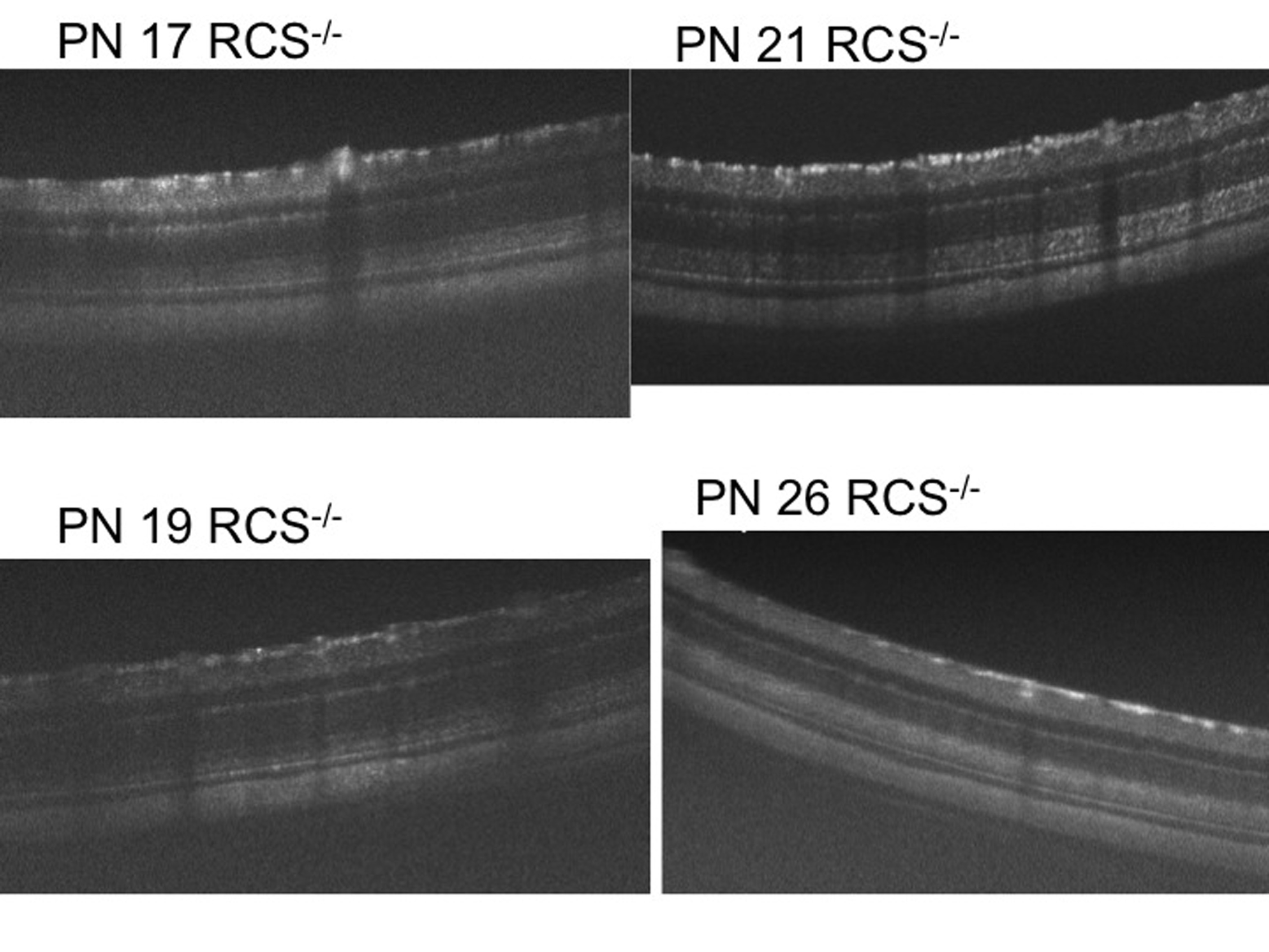

Supplement: S2 Fig — (TIF) [file pone.0162835.s002.tif]

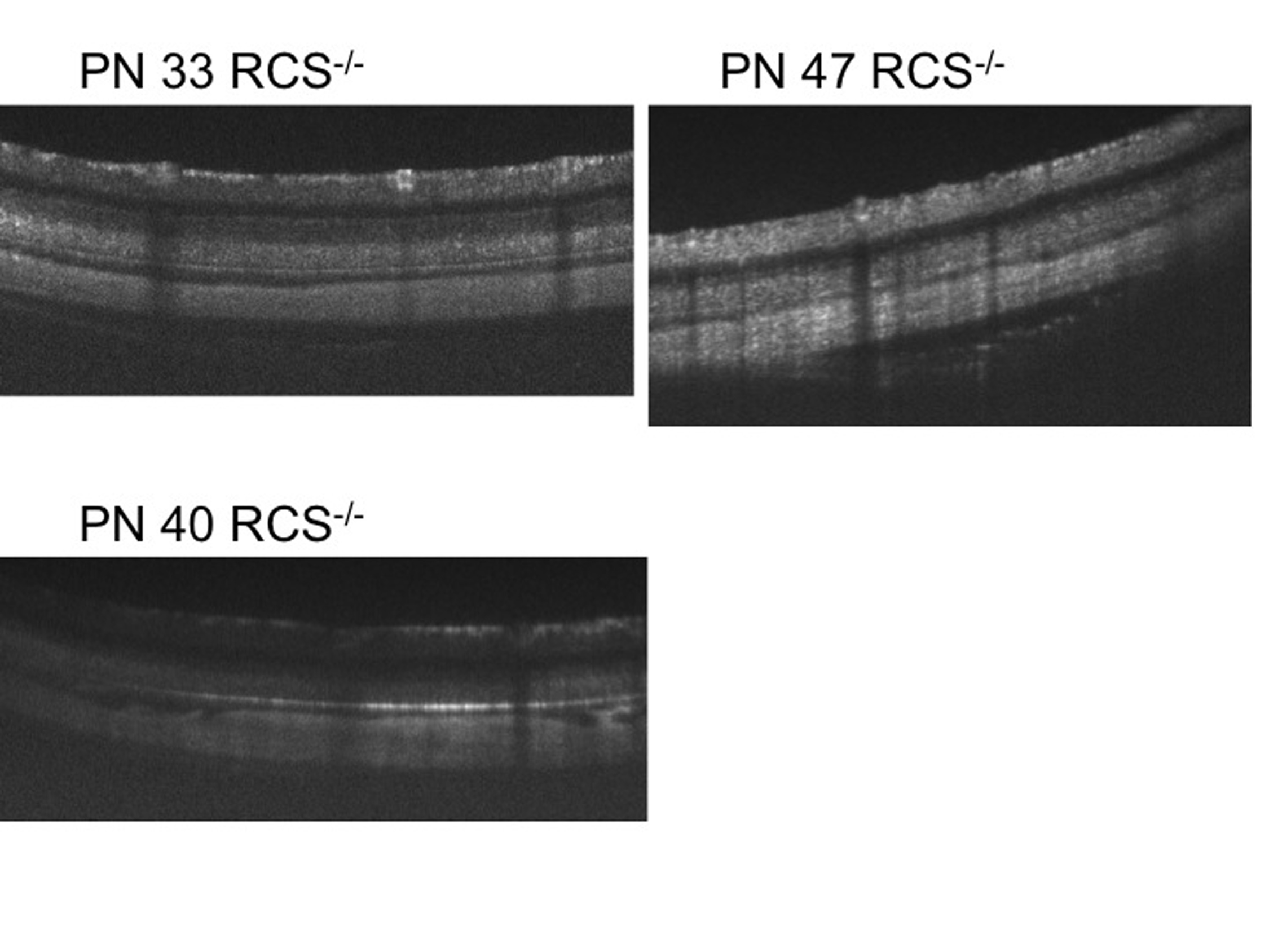

Supplement: S3 Fig — (TIF) [file pone.0162835.s003.tif]
