# Supplementary material for: Optical Coherence Tomography of Retinal Degeneration in Royal College of Surgeons Rats and Its Correlation with Morphology and Electroretinography
Source: PLoS One. 2016 Sep 19;11(9):e0162835. doi: 10.1371/journal.pone.0162835 (PMC5028068; doi:10.1371/journal.pone.0162835)
Supplement: S1 Table — Length: μm. (PDF) [file pone.0162835.s004.pdf]

RCS rat retinal layer summary 1

	inner layer (A)	outer layers (B + C)	RPE + choroid (D)		inner layer (A)	outer layers (B + C)	RPE + choroid (D)
wild RCS				dystrophic RCS 17 do			
18 do III-L	101.766	121.2298	39.9463	17 do I-R	92.6855	125.4551	36.1255
18 do II-L	95.1298	119.0636	33.7815	17 do II-L	89.9628	151.8838	41.1777
18 do II-R	103.6255	121.1034	28.8116	17 do II-R	98.0862	135.1185	37.0625
18 do IX-L	104.1462	113.7618	31.3949	17 do III-L	107.3397	141.4715	35.9376
18 do X-R	105.8307	117.6998	28.9304	17 do III-R	96.6355	135.6682	31.5032
mean ± SD	102.0996 ± 4.1572	118.5717 ± 3.0664	32.5729 ± 4.5997	mean ± SD	96.9419 ± 6.640	137.9194 ± 9.6940	36.3613 ± 3.4470
					P = 0.179	P = 0.003	P = 0.179
wild RCS				dystrophic RCS			
19 do I-L	109.2746	123.9205	35.6372	19 do I-L	99.0311	140.481	34.3998
19 do III-R	105.5006	115.6957	36.1776	19 do II-L	100.1051	127.5541	38.0763
19 do IX-L	89.0444	94.3264	27.7866	19 do II-R	100.2364	129.7418	36.4268
19 do IX-R	105.0683	118.3003	33.3452	19 do IV-R	100.6599	132.2744	33.4918
19 do VII-L	104.2124	111.9226	30.2983	19 do V-L	97.5604	123.0284	35.0553
mean ± SD	102.6201 ± 7.8328	112.8331 ± 11.2288	32.6490 ±3.5719	mean ± SD	99.5186 ± 1.2484	130.6159 ± 6.47649	35.4900 ± 1.7984
					P = 0.407	P = 0.015	P = 0.151
wild RCS				dystrophic RCS			
25 do I-R	89.6031	109.5836	34.0233	21 do I-R	100.426	133.0220	30.3879
25 do II-R	89.5737	115.202	37.22	21 do II-R	96.1578	125.0280	39.8458
25 do IV-L	93.4860	112.0236	32.0536	21 do III-L	109.1551	141.5433	33.2058
25 do V-L	94.7965	122.2583	35.0885	21 do III-R	103.7144	141.6617	32.0013
25 do VIII-R	89.7680	112.9932	31.8012	21 do IV-L	103.4137	130.0389	34.4279
mean ± SD	91.4455 ± 2.5052	114.4121 ± 4.8275	34.0373 ± 2.2456	mean ± SD	102.5734 ± 4.7723	134.2588 ± 7.2872	33.9737 ± 3.6065
					P = 0.991	P = 0.007	P = 0.576
wild RCS				dystrophic RCS			
32 do II-L	88.4746	102.6927	24.0982	26 d0 I-R	83.3411	126.3164	31.7875
32 do II-R	82.7879	106.9510	25.506	26 do III-L	88.8071	133.3935	34.4213
32 do IV-L	88.5051	106.951	42.3426	26 do IV-R	93.0230	136.4043	35.1246
32 do V-R	79.5578	113.8525	32.2383	26 do V-L	92.6931	127.4538	38.1320
32 do VII-L	85.3785	114.9050	27.1869	26 do V-R	103.3975	122.2704	34.1180
mean ± SD	84.9408 ± 3.8404	109.0704 ± 5.1616	30.2744 ± 7.4148	mean ± SD	92.2524 ± 7.3533	129.1677 ± 5.6760	34.7167 ± 2.2835
					P = 0.822	P = 0.002	P = 0.648
				dystrophic RCS			
wild RCS 39 do				33 do I-L	97.4790	118.0539	32.3110
39 do II-L	88.4716	102.6924	24.0953	33 do I-R	98.9022	113.8223	28.6273
39 do II-R	82.7879	106.9510	25.5060	33 do II-R	83.1033	104.1141	37.5241
39 do IV-L	88.5032	106.8854	42.3426	33 do III-L	97.2927	106.7769	40.9090
39 do V-R	79.5578	113.8525	32.2383	33 do VII-R	100.0248	117.1559	28.2123
39 do VII-L	85.3785	114.905	27.1869	mean ± SD	95.3604 ± 6.9419	111.9846 ± 6.2454	33.5167 ± 5.5723
mean ± SD	84.9398 ± 3.8393	109.0573 ± 5.1685	30.2738 ± 7.4153		P = 0.019	P = 0.444	P = 0.457
				RCS -/- 37 do			
wild RCS 46 do				37 do I-L	94.6480	92.9777	34.1390
46 do I-L	94.2822	107.2353	24.4082	37 do I-R	97.7367	86.7492	33.7172
46 do I-R	90.6892	104.0366	31.9194	37 do II-L	90.6489	88.3128	57.2406
46 do II-L	85.9062	108.4627	25.1472	37 do III-R	101.9608	79.8595	35.0038
46 do IV-R	85.9119	107.5236	31.0052	37 do III-L	93.7924	84.1653	31.3947
46 do VIII-R	82.2806	103.4428	27.9710	mean ± SD	95.7574 ± 4.2820	86.4129 ± 4.8687	38.2991 ± 10.6725
mean ± SD	87.8140 ± 4.6899	106.1402 ± 2.2477	28.0902 ± 3.3687		P = 0.003	P < 0.001	P = 0.205
				dystrophic RCS			
wild RCS 53 do				~~40 do I-R~~	~~99.6498~~	~~35.8439~~	~~40.3989~~
53 do II-L	81.2696	105.2545	32.6866	40 do II-R	91.3336	68.0888	35.1155
53 do IV-L	82.0151	112.9623	33.6500	40 do III-R	96.7971	92.7553	33.1416
53 do IX-L	82.6917	108.6079	37.4239	40 do IV-L	98.5336	86.4058	26.4042
53 do VI-L	82.6857	108.0417	33.8302	40 do IV-R	91.7360	85.8195	31.1077
53 do VII-L	88.3984	110.3806	32.9000	40 do I-R agair	83.7445	52.6959	38.4746
mean ± SD	83.4121 ± 2.8432	109.0494 ± 2.8590	34.0981 ± 1.9210	mean ± SD	92.4290 ± 5.7760	77.1531 ± 16.4656	32.8487 ± 4.51214
					P = 0.042	P = 0.003	P = 0.526
wild RCS 67do				dystrophic RCS			
67 do I-R	81.6838	108.1121	33.0393	47 do II-L	98.4242	58.0378	37.4542
67 do IX-R	84.2936	111.9290	37.1074	47 do II-R	104.7529	68.5085	30.2362
67 do VI-L	80.7689	103.8693	29.0784	47 do IV-L	117.0197	42.9570	28.8929
67 do VI-R	87.7981	103.8540	27.6007	47 do V-R	104.5243	50.9597	34.0765
67 do X-L	82.3062	111.4266	29.2277	47 do VI-L	98.6864	66.6260	27.9094
mean ± SD	83.3701 ± 2.7929	107.8382 ± 3.9151	31.2107 ± 3.8610	mean ± SD	104.6815 ± 7.5392	57.4178 ± 10.7122	31.7138 ± 3.9733
					P = 0.003	P < 0.001	P = 0.158
				dystrophic RCS			
				54 do I-R	100.7152	40.7036	31.7593
				54 do III-L	97.1867	45.4672	30.6741
				54 do IV-L	102.0165	43.9360	34.2131
				54 do V-L	95.3092	48.2603	34.4636
				54 do VI-L	103.7419	46.3301	32.5208
				mean ± SD	99.7939 ± 3.4739	44.9394 ± 2.8368	32.7262 ± 1.6138
					P < 0.001	P < 0.001	P = 0.256
				dystrophic RCS			
				61 do III-L	105.3505	34.2375	32.5911
				61 do III-R	90.0257	43.6263	29.0691
				61 do V-L	101.6598	35.5014	27.4405
				61 do V-R	90.5333	43.6527	30.4516
				61 do VI-R	90.5333	43.6527	30.4516
				mean ± SD	95.6205 ± 7.3180	40.1341 ± 4.8267	30.0008 ± 1.9065

	P = 0.008	P < 0.001	P = 0.547
dystrophic RCS			
74 do I-L	98.0717	34.9029	28.6587
74 do I-R	104.2755	43.1879	26.2167
74 do II-L	86.0497	35.4806	23.6632
74 do II-R	90.0141	31.9559	23.1233
74 do III-L	97.9014	35.5082	30.536
mean ± SD	95.2625 ± 7.2200	36.2071 ± 4.1690	26.4396 ± 3.1804
	P =0.018	P < 0.001	P = 0.066
dystrophic RCS			
111 do I-L	83.5302	35.1869	21.0095
111 do I-R	95.8078	35.7469	28.5007
111 do II-L	93.2379	34.4361	26.7744
111 do II-R	95.8078	35.7469	28.4603
111 do III-R	90.4609	30.0961	24.0764
mean ± SD	91.7689 ± 5.1081	34.2426 ± 2.3795	25.7643 ± 3.261
	P = 0.012	P < 0.001	P = 0.042
