# Supplementary material for: Optical Coherence Tomography of Retinal Degeneration in Royal College of Surgeons Rats and Its Correlation with Morphology and Electroretinography
Source: PLoS One. 2016 Sep 19;11(9):e0162835. doi: 10.1371/journal.pone.0162835 (PMC5028068; doi:10.1371/journal.pone.0162835)
Supplement: S2 Table — Length: μm. (PDF) [file pone.0162835.s005.pdf]

RCS rat retinal thickness analyses

	OPL and ONL (B)	IS and OS (C)		OPL and ONL (B)	IS and OS (C)		OPL and ONL (B)	IS and OS (C)
RCS wild 18 do			RCS−/− 19 do	P = 0.731	P = 0.003	RCS−/− 17 do	P = 0.347	P < 0.001
18 do Ⅱ−L	88.9596	30.065	19 do Ⅱ−R	88.4968	43.2202	17 do Ⅰ−R	85.8619	40.3075
18 do Ⅱ−L	88.0101	30.7151	19 do Ⅲ−L	89.3291	42.2564	17 do Ⅱ−L	107.0373	50.1699
18 do Ⅲ−L	87.0702	34.4728	19 do Ⅲ−R	88.364	30.5697	17 do Ⅱ−R	89.4052	45.7133
18 do Ⅷ−R	90.2336	30.7310	19 do Ⅳ−R	113.7683	46.4371	17 do Ⅲ−L	92.0065	49.4650
18 do Ⅹ−R	89.7687	29.3428	19 do Ⅶ−R	84.2538	41.4659	17 do Ⅲ−R	88.6337	44.9652
mean ± SD	88.8094 ± 1.2879	31.0653 ± 1.9883	mean ± SD	92.8424 ± 11.8632	40.7899 ± 6.0178	mean ± SD	92.5889 ± 8.3685	46.1242 ± 3.9647
RCS wild 19 do			RCS−/− 21 do	P = 0.095	P = 0.006			
19 do Ⅰ−L	91.9936	30.0369	21 do Ⅰ−L	87.7293	57.3709			
19 do Ⅲ−L	95.6438	29.1787	21 do Ⅰ−R	80.0826	52.9394			
19 do Ⅲ−R	88.364	30.5697	21 do Ⅱ−R	77.2848	47.7432			
19 do Ⅸ−R	92.3098	27.6481	21 do Ⅲ−R	85.2061	56.4556			
19 do Ⅶ−L	86.0149	25.8157	21 do Ⅳ−R	80.6016	49.4373			
mean ± SD	90.8652 ± 3.7409	28.8652 ± 1.9317	mean ± SD	82.1809 ± 4.2073	52.7893 ± 4.2177			
RCS wild 25 do			RCS −/− 26 do	P = 0.025	P < 0.001			
25 do Ⅱ−L	78.185	30.8582	26 do Ⅰ−R	76.2667	50.6097			
25 do Ⅱ−R	85.9574	30.5041	26 do Ⅲ−R	77.8197	50.4181			
25 do Ⅴ−L	90.0526	30.4683	26 do Ⅳ−R	80.2828	56.1215			
25 so Ⅶ−R	81.495	37.0110	26 do Ⅴ−L	71.4271	56.0267			
25 do Ⅷ−R	80.5209	33.9321	26 do Ⅴ−R	67.6361	54.6343			
mean ± SD	83.2422 ± 4.7378	32.5547 ± 2.8806	mean ± SD	74.6865 ± 5.0982	53.5621 ± 2.8450			
RCS wild 32 do			RCS−/− 33 do	P < 0.001	P < 0.001			
32 do Ⅰ−L	74.2008	32.8322	33 do Ⅰ−R	56.8090	57.0133			
32 do Ⅳ−R	85.5279	34.3548	33 do Ⅱ−R	57.0702	54.5343			
32do Ⅴ−R	78.9237	35.9458	33 do Ⅲ−L	60.2915	48.7437			
32 do Ⅶ−L	88.2271	39.5019	33 do Ⅴ−R	54.2448	46.912			
32 do Ⅹ−R	80.2666	34.5626	33 do Ⅵ−L	47.3937	52.9157			
mean ± SD	81.4292 ± 5.5428	35.4395 ± 2.5250	mean ± SD	55.1618 ± 4.8439	52.0238 ± 4.1498			
RCS wild 39 do			RCS−/− 40 do	P < 0.001	P = 0.033	RCS−/− 37 do	P < 0.001	P < 0.001
39 do Ⅱ−L	72.8161	30.1763	40 do Ⅰ−R	21.8038	31.4521	37 do Ⅰ−L	28.7938	69.8720
39 do Ⅱ−R	77.0718	29.8792	40 do Ⅱ−R	24.8595	43.2193	37 do Ⅰ−R	28.6127	58.3174
39 do Ⅳ−L	76.2118	30.6736	40 do Ⅲ−R	38.9360	53.4123	37 do Ⅱ−L	29.5900	58.7228
39 do Ⅴ−R	81.2770	34.3933	40 do Ⅳ−L	29.5216	57.2504	37 do Ⅲ−R	34.8465	61.3046
39 do Ⅶ−L	77.3385	38.9102	40 do Ⅳ−R	25.2133	59.8146	37 do Ⅲ−L	28.1314	51.4359
mean ± SD	76.9430 ± 3.0217	32.8065 ± 3.8558	mean ± SD	28.0668 ± 6.6692	49.0297 ± 11.6815	mean ± SD	29.9949 ± 2.7626	59.9305 ± 6.6510
RCS wild 46 do			RCS−/− 47 do	P < 0.001	P = 0.003			
46 do Ⅰ−L	67.9220	39.9499	47 do Ⅱ−L	19.4487	38.5891			
46 do Ⅰ−R	73.1216	31.2327	47 do Ⅱ−R	14.5891	54.9194			
46 do Ⅱ−L	78.3478	30.4838	47 do Ⅲ−R	17.8682	58.2742			
46 do Ⅳ−R	80.8881	33.6173	47 do Ⅵ−L	19.1494	47.4766			
46 do Ⅷ−L	74.0425	33.5613	47 do Ⅵ−R	19.1494	47.4766			
mean ± SD	74.8644 ± 5.0082	33.7690 ± 3.7248	mean ± SD	18.0410 ± 2.0239	49.3472 ± 7.6400			
RCS wild 54 do			54 do		P < 0.001, P < 0.001			
54 do Ⅱ−L	73.5378	30.4256	54 do Ⅰ−R	undetectable = 0	40.7036			
54 do Ⅳ−L	78.8150	35.8677	54 do Ⅲ−L		45.4672			
54 do Ⅸ−L	74.2752	34.9717	54 do Ⅳ−L		43.9360			
54 do Ⅳ−L	74.0487	33.9930	54 do Ⅴ−L		48.2603			
	76.6847	33.6736	54 do Ⅵ−L		46.3301			
mean ± SD	75.4723 ± 2.2274	33.7863 ± 2.0668	mean ± SD		44.9394 ± 2.8368			
RCS wild 67 do			61 do		P = 0.048, P = 9.039, P = 0.090 (67 doとの比較)			
67 do Ⅰ−R	77.1593	30.9528	61 do Ⅲ−L	undetectable = 0	34.2375			
67 do Ⅸ−R	73.2758	38.6532	61 do Ⅲ−R		43.6263			
67 do Ⅵ−L	70.1169	35.0841	61 do Ⅴ−L		35.5014			
67 do Ⅵ−R	69.5930	35.4667	61 do Ⅴ−R		43.6527			
67 do Ⅹ−L	76.1385	36.4057	61 do Ⅵ−R		43.6527			
mean ± SD	73.2567 ± 3.4211	35.3125 ±2.8035	mean ± SD		40.1341 ± 4.8267			
			74 do		P = 0.358 P = 0.278 P = 0.701			
			74 do Ⅰ−L	undetectable = 0	34.9029			
			74 do Ⅰ−R		43.1879			
			74 do Ⅱ−L		35.4806			
			74 do Ⅱ−R		31.9559			
			74 do Ⅲ−L		35.5082			
			mean ± SD		36.2071 ± 4.1690			
			111 do		P = 0.817 P =0.785			
			111 do Ⅰ−L	undetectable = 0	35.1869			
			111 do Ⅰ−R		35.7469			
			111 do Ⅱ−L		34.4361			
			111 do Ⅱ−R		35.7469			
			111 do Ⅲ−R		30.0961			
			mean ± SD		34.2426 ± 2.3795			
