# Supplementary material for: Optical Coherence Tomography of Retinal Degeneration in Royal College of Surgeons Rats and Its Correlation with Morphology and Electroretinography
Source: PLoS One. 2016 Sep 19;11(9):e0162835. doi: 10.1371/journal.pone.0162835 (PMC5028068; doi:10.1371/journal.pone.0162835)
Supplement: S3 Table — A-wave and B-wave: μV. (PDF) [file pone.0162835.s006.pdf]

RCS+/+ rat ERG A and B waves

		Sel	B-wave	T(B)	A-wave	T(A)	G-Flash	UV-Flash	G-Delay	UV-Delay	G-CBL	UV-CBL	Delay BS	Notes	NAME
15 days old		Yes	170.479	85.8	−101.379	20	3	0	0	0	0	0	10	10	RCS++15do#1R
	1														
17 days old		Yes	232.184	71.8	−152.716	20	3	0	0	0	0	0	10	1	RCS++17do#1R
	2	Yes	234.898	73.2	−130.303	21.4	3	0	0	0	0	0	10	4	RCS++17do#1L
		Yes	205.485	78.2	−116.67	17.4	3	0	0	0	0	0	10	10	RCS++17do#2L
		Yes	154.054	83.8	−84.005	29	3	0	0	0	0	0	10	13	RCS++17do#2ROK
24 days old		Yes	98.567	74.4	−46.035	17.2	3	0	0	0	0	0	10	1	RCS++24do#1R
	3	Yes	233.359	70	−131.473	19	3	0	0	0	0	0	10	4	RCS++24do#1L
		Yes	161.952	73.8	−98.851	14.6	3	0	0	0	0	0	10	7	RCS++24do#2R
		Yes	235.321	82.4	−131.687	17	3	0	0	0	0	0	10	10	RCS++24do#2L
31 days old		Yes	125.297	71.4	−69.669	16.6	3	0	0	0	0	0	10	1	RCS++31do#1R
	4	Yes	109.293	79.8	−62.751	17.8	3	0	0	0	0	0	10	4	RCS++31do#1L
		Yes	115.649	70	−83.569	15.2	3	0	0	0	0	0	10	7	RCS++31do#2R
		Yes	152.783	73.8	−113.726	15.8	3	0	0	0	0	0	10	10	RCS++31do#2L
37 days old		Yes	116.928	70	−90.59	14.8	3	0	0	0	0	0	10	1	RCS+/+ 37 do R−1−3
	5	Yes	99.531	70	−77.425	16	3	0	0	0	0	0	10	4	RCS+/+ 37 do L−1−3
		Yes	175.256	75.8	−113.887	14.6	3	0	0	0	0	0	10	8	RCS+/+ 37 do R−2−3
		Yes	162.82	70	−84.345	17.2	3	0	0	0	0	0	10	11	RCS+/+ 37 do L−2−3
52 days old		Yes	132.958	70	−78.062	15.2	3	0	0	0	0	0	10	1	RCS+/+ 52do R−1−3
	6	Yes	108.507	70	−75.849	15	3	0	0	0	0	0	10	4	RCS+/+ 52do L−1−3
		Yes	218.623	70	−129.516	16.6	3	0	0	0	0	0	10	7	RCS+/+ 52do R−2−3
