# Supplementary material for: Optical Coherence Tomography of Retinal Degeneration in Royal College of Surgeons Rats and Its Correlation with Morphology and Electroretinography
Source: PLoS One. 2016 Sep 19;11(9):e0162835. doi: 10.1371/journal.pone.0162835 (PMC5028068; doi:10.1371/journal.pone.0162835)
Supplement: S4 Table — A-wave and B-wave: μV. (PDF) [file pone.0162835.s007.pdf]

RCS-/- rat ERG A & B waves

	Sel	B-wave	T(B)	A-wave	T(A)	G-Flash	UV-Flash	G-Delay	UV-Delay	G-CBL	UV-CBL	Delay BS	Notes	NAME
17 days old	Yes	196.794	74.6	-122.904	23.6	3	0	0	0	0	0	10	1	RCS-/-17do, R-1-3
7	Yes	205.884	70	-92.103	23.4	3	0	0	0	0	0	10	5	RCS-/-17do, L-1-3
	Yes	152.203	73	-61.842	24.4	3	0	0	0	0	0	10	8	RCS-/-17do, R-2-3
	Yes	203.764	78.4	-96.207	30	3	0	0	0	0	0	10	12	RCS-/-17do, L-2-3
	Yes	159.182	70.2	-82.361	21	3	0	0	0	0	0	10	15	RCS-/-17do, R-3-3
	Yes	177.391	83	-88.36	26.4	3	0	0	0	0	0	10	18	RCS-/-17do, L-3-3
19 days old	Yes	54.609	72.8	-29.818	22.8	3	0	0	0	0	0	10	12	RCS-/-19do L-1-3
8	Yes	113.664	80.4	-50.635	32	3	0	10	0	0	0	10	15	RCS-/-19do R-1-3
	Yes	109.224	84.6	-46.116	34.8	3	0	0	0	0	0	10	19	RCS-/-19do R-2-3
	Yes	122.454	70	-43.328	22	3	0	0	0	0	0	10	23	RCS-/-19do L-2-3
22 days old	Yes	135	56	-44.09	19	3							1	RCS-/-22do R-1-3
	Yes	220	62.8	-90	26.6	3							4	RCS-/-22do L-1-3
	Yes	132.995	73.6	-56.611	22.4	3							7	RCS-/-22do R-2-3
	Yes	135.915	84.2	-45	38.8	3							10	RCS-/-22do L-2-3
23 days old	Yes	105.209	70.5	-32.189	26	3							30	RCS-/-22do R-2-3
	Yes	151.665	83.8	-55.795	27.6	3							33	RCS-/-22do L-2-3
	Yes	53.705	149.8	-50.413	34.2	3							36	RCS-/-22do R-3-3
28 days old	Yes	57.234	70.2	-14.602	38.6	3	0	0	0	0	0	10	1	RCS-/-28do#1R
9	Yes	59.033	70	-29.215	2.8	3	0	0	0	0	0	10	4	RCS-/-28do#1L
	Yes	100.724	70.8	-27.929	22	3	0	0	0	0	0	10	8	RCS-/-28do#2R
	Yes	119.955	76.4	-30.687	22.4	3	0	0	0	0	0	10	11	RCS-/-28do#2L
29 days old	Yes	72.608	75.2	-17.607	25.6	3	0	0	0	0	0	10	1	RCS-/- 29do#1R
10	Yes	93.888	82.2	-15.213	1.8	3	0	0	0	0	0	10	5	RCS-/- 29do#1L
33 days old	Yes	39.952	87	-11.864	42.8	3	0	0	0	0	0	10	9	RCS--33do#1R
11	Yes	44.581	109.2	-3.34	39.4	3	0	0	0	0	0	10	12	RCS--33do#1L
	Yes	53.051	80.8	-14.922	18.2	3	0	0	0	0	0	10	20	RCS--33do#2L
	Yes	31.578	80.8	-1.335	14.6	3	0	0	0	0	0	10	16	RCS--33do#2R
	Yes	19.996	104.4	-4.889	14.8	3	0	0	0	0	0	10	23	RCS--33do#3R
	Yes	34.116	74	-12.522	37.6	3	0	0	0	0	0	10	26	RCS--33do#3L
42 days old	Yes	21.142	83.2	-5.541	10.4	3	0	0	0	0	0	10	1	RCS--42do#1R
12	Yes	0.096	71.8	-7.485	7.4	3	0	0	0	0	0	10	4	RCS--42do#1L
	Yes	24.979	70	-8.442	0	3	0	0	0	0	0	10	7	RCS--42do#2R
46 days old	Yes	6.478	73.8	-27.202	51.6	3	0	10	0	0	0	10	13	RCS--46do#1R
13	Yes	10.548	88	-19.318	48	3	0	0	0	0	0	10	16	RCS--46do#1L
	Yes	40.914	91	-13.65	16.6	3	0	0	0	0	0	20	19	RCS--46do#2R
	Yes	20.814	87.2	-3.714	12.8	3	0	0	0	0	0	10	22	RCS--46do#2L
53 days old	Yes	3.408	78.2	0.608	12.4	3	0	0	0	0	0	10	13	RCS--53do#1R
14	Yes	12.164	149.2	-13.672	59.8	3	0	0	0	0	0	10	16	RCS--53do#1L
	Yes	11.247	70	-15.084	0	3	0	0	0	0	0	10	19	RCS--53do#2R
	Yes	0.169	79.6	-0.222	9.6	3	0	0	0	0	0	10	22	RCS--53do#2L
