# Supplementary material for: Optical Coherence Tomography of Retinal Degeneration in Royal College of Surgeons Rats and Its Correlation with Morphology and Electroretinography
Source: PLoS One. 2016 Sep 19;11(9):e0162835. doi: 10.1371/journal.pone.0162835 (PMC5028068; doi:10.1371/journal.pone.0162835)
Supplement: S5 Table — A-wave and B-wave: μV. (PDF) [file pone.0162835.s008.pdf]

ERG data summary

No	PN day	B wave amplitudes	B wave latent time	A wave amplitudes	A wave latent time
	RCS+/+				
1	15	170.48	85.8	−101.38	20
2	17	206.65 ± 37.50 (18.75)	76.75 ± 5.44 (2.72)	−120.92 ± 28.75 (14.38)	21.95 ± 4.98 (2.49)
3	24	182.30 ± 65.43 (32.71)	75.15 ± 5.21 (2.61)	−102.01 ± 40.38 (20.19)	16.95 ± 1.18 (0.90)
4	31	125.76 ± 19.18 (9.59)	73.75 ± 4.32 (2.16)	−82.43 ± 22.59 (11.29)	16.35 ± 1.12 (0.56)
5	37	138.63 ± 36.17 (18.09)	71.45 ± 2.90 (1.45)	−91.56 ±15.82 (7.91)	15.65 ± 1.20 (0.60)
6	52	153.36 ± 57.82 (33.38)	70.00 ± 0.00 (0.00)	−94.48 ± 30.04 (17.53)	15.60 ± 0.87 (0.50)
	RCS−/−				
7	17	182.53 ± 23.20 (9.47)	74.87 ± 5.05 (2.06)	−90.63 ± 19.89 (8.12)	24.80 ± 3.09 (1.26)
8	19	99.99 ± 30.75 (15.37)	76.95 ± 6.73 (3.37)	−42.47 ± 8.96 (4.48)	27.90 ± 6.46 (3.23)
9	22	155.98 ± 42.70 (21.35)	69.15 ± 12.38 (6.19)	−58.93 ± 21.49 (10.74)	26.70 ± 8.64 (4.32)
9	23	103 26 ± 49.00 (28.29)	101.37 ± 42.46 (24.53)	−46.13 ± 12.37 (7.14)	29.27 ± 4.35 (2.51)
9	28	84.24 ± 31.16 (15.58)	71.85 ± 3.05 (1.53)	−25.61 ± 7.42 (3.71)	21.45 ± 14.64 (7.32)
10	29	78.70 ± 4.95 (3.50)	78.70 ± 4.95 (3.50)	−16.41 ± 1.69 (1.19)	13.70 ± 16.83 (11.90)
11	33	89.37 ± 14.20 (5.80)	89.37 ± 14.20 (5.80)	−8.15 ± 5.64 (2.30	27.90 ± 13.35 (5.45)
12	42	75.00 ± 7.16 (4.13)	75.00 ± 7.16 (4.13)	−7.16 ± 1.48 (0.85)	5.93 ± 5.35 (3.09)
13	46	32.25 ± 20.38 (10.19)	32.25 ± 20.38 (10.19)	−15.97 ± 9.88 (4.94)	32.25 ± 20.38 (10.19
14	53	20.24 ±26.76 (13.38)	20.45 ± 26.76 (13.38)	−7.09 ± 8.44 (4.22)	20.45 ± 26.77 (13.38)

mean ± SD (SE)

All data can be regarded as normal distribution.

Statistical Analyses

RCS−/−	a−wave	b−wave
ANOVA	P < 0.001	P < 0.001
Bonferroni	17:19 P < 0.001	17:19 P = 0.003
	17:22 P = 0.157	17:22 P = 1.000
	17:23 P = 0.002	17:23 P = 0.014
	17:28 P < 0.001	17:28 P < 0.001
	17:29 P < 0.001	17:29 P < 0.001
	17:33 P < 0.001	17:33 P < 0.001
	17:42 P < 0.001	17:42 P < 0.001
	17:46 P < 0.001	17:46 P < 0.001
	17:53 P < 0.001	17:53 P < 0.001

when RCS+/+ 17do is included,

ANOVA	P < 0.001	
	1:17 P = 0.254	1:17 P = 1.000
	1:19 P < 0.001	1:19 P < 0.001
	1:22 P < 0.001	1:22 P = 1.000
	1:23 P < 0.001	1:23 P = 0.002
	1:28 P < 0.001	1:28 P < 0.001
	1:29 P < 0.001	1:29 P = 0.001
	1:33 P < 0.001	1:33 P < 0.001
	1:42 P < 0.001	1:42 P < 0.001
	1:46 P < 0.001	1:46 P < 0.001
	1:53 P < 0.001	1:53 P < 0.001

RCS+/+		
ANOVA	P = 0.559	P = 0.202
